# Elevated glypican‐1 expression is associated with an unfavorable prognosis in pancreatic ductal adenocarcinoma

**DOI:** 10.1002/cam4.1064

**Published:** 2017-04-24

**Authors:** Haizhen Lu, Fangfei Niu, Fang Liu, Jiajia Gao, Yulin Sun, Xiaohang Zhao

**Affiliations:** ^1^ Department of PathologyNational Cancer Center/Cancer HospitalChinese Academy of Medical Science & Peking Union Medical CollegeBeijing100021China; ^2^ State Key Laboratory of Molecular OncologyNational Cancer Center/Cancer HospitalChinese Academy of Medical Sciences & Peking Union Medical CollegeBeijing100021China

**Keywords:** Biomarker, gene expression, glypican‐1, pancreatic ductal adenocarcinoma, prognostic factor

## Abstract

Pancreatic ductal adenocarcinoma (PDAC) is the most lethal cancer in humans, with a 5‐year survival rate of <5%. Recently, glypican‐1 (GPC1)‐expressing circulating exosomes were found to be a promising diagnostic tool for PDAC. However, the aberrant expression of GPC1 has not been systematically evaluated in large‐scale clinical samples of PDAC. Here, we performed a comprehensive analysis of GPC1 mRNA and protein expression features. Included in this study were 178 PDAC patients from the cancer genome atlas (TCGA) and 186 subjects whose tissues were used in immunohistochemical staining assays. We demonstrated that GPC1 mRNA was silenced in normal pancreata; however, it was re‐expressed in PDAC tissues probably because of the promoter hypomethylation. The GPC1 protein was barely expressed in the normal and adjacent noncancerous pancreata. In tumor tissues, 59.7% (111/186) of the detected samples showed positive expression. Notably, GPC1 was elevated in 63.6% (34/55) of early stage cases. High levels of GPC1 were associated with poorer differentiation and larger tumor diameters. Kaplan–Meier analysis showed a significant difference in overall survival between the groups categorized by GPC1 expression (*P* = 0.0028). Multivariate analyses indicated that GPC1 was a significant risk factor for poor overall survival with a 1.82‐fold increase in the hazard ratio (*P* = 0.0022). In conclusion, during pancreatic tumorigenesis, GPC1 was ectopically expressed and served as an independent poor prognostic factor. Our findings highlighted the alluring prospect of GPC1 as an early diagnostic and prognostic marker as well as a therapeutic target for PDAC.

## Introduction

Pancreatic cancer is the most lethal cancer in humans worldwide. Although its incidence is ranked 15th among common malignancies 1, only approximately 4% of patients live 5 years after diagnosis 2. Notably, due to the asymptomatic nature of early‐stage pancreatic cancer, approximately 80–85% of cases at initial diagnosis present with unresectable advanced or metastatic disease. The median survival time for these patients is only 3–14 months 2. Therefore, screening or early detection of pancreatic cancer is a promising tool to improve clinical outcome.

Today, carbohydrate antigen 19‐9 (CA 19‐9) is a commonly utilized tumor marker in the diagnosis and monitoring of pancreatic ductal adenocarcinoma (PDAC), the most common form of pancreatic neoplasm. It is mainly synthesized by normal pancreatic and biliary ductal cells and, to a lesser extent, by gastric, colonic, endometrial, and salivary epithelia 3. Approximately 10% of Caucasian and 22% of non‐Caucasian population do not express CA 19‐9 due to the lack of the fucosyltransferase needed for CA 19‐9 production 3, 4. In 16% of PDAC cases, CA19‐9 was elevated 2–3 years prior to diagnosis, with sensitivity increasing toward diagnosis 5. In early‐stage (stage I and II) pancreatic cancer, up to 66% of patients had an elevated CA19‐9 level at diagnosis 4, and the diagnostic sensitivity and specificity were 55–75% and 56–81%, respectively 6. Overall, CA 19‐9 is neither a very sensitive nor specific marker for resectable or local PDAC. Therefore, identifying novel serum markers that could aid in the detection of early‐stage PDAC remains a clear unmet need.

Recently, it was shown that glypican‐1‐expressing (GPC1^+^) circulating exosomes (crExos) can be used to detect early‐stage PDAC and gain insights into disease progression and tumor burden 7, 8. GPC1^+^ crExos levels were significantly elevated in patients with histologically confirmed pancreatic cancer precursor lesions and PDAC compared with those in patients with benign pancreatic disease and healthy controls. Importantly, GPC1^+^ crExos showed a nearly 100% sensitivity and specificity even for early stage PDAC, indicating its outstanding potential for early detection of the disease 7.

GPC1 is a heparan sulfate proteoglycan (HSPG) that binds to the external surface of the plasma membrane by a glycosyl‐phosphatidylinositol (GPI) anchor 9, 10. The human genome includes six glypicans (GPC1 to GPC6), and GPC1 is relatively closely related to GPC2 with approximately 37% amino‐acid identity. Under physiological conditions, GPC1 is expressed predominantly in the brain, skin, skeletal muscle, kidney and testis, but there is little or no expression in the liver, lung, pancreas and blood cells during development 11. In PDAC, it was reported that GPC1 was significantly overexpressed at both the mRNA and protein levels 12, 13. Moreover, increased GPC1 was associated with perineural invasion and poor prognosis of PDAC based on limited samples 13. However, the aberrant expression of GPC1 and its clinical significance in PDAC have not been evaluated in large‐scale clinical samples. In this study, we analyzed the mRNA and protein expression characteristics of GPC1 in PDAC using the RNA sequencing dataset from the cancer genome atlas (TCGA) and by immunohistochemical staining assays.

## Materials and Methods

### TCGA RNA sequencing data mining

The RNA sequencing data from 178 patients with pancreatic adenocarcinoma were obtained from TCGA (https://tcga-data.nci.nih.gov). The subjects included eight pancreatic neuroendocrine carcinoma patients, four pancreatic mucinous noncystic carcinoma patients, and 13 patients with an unknown pathological type; the remaining patients were ductal (*n* = 137) or another type of (*n* = 16) pancreatic adenocarcinoma. The final cohort used in this study excluded the eight patients with pancreatic neuroendocrine carcinoma because these cases originated from the endocrine portion of the pancreas. Among the 170 enrolled patients, 157 cases had detailed clinical information. The median duration of patient follow‐up was 13 months. The expression of *GPC1* mRNA and its clinical significance were analyzed in these patients.

### Tissue microarrays

Four tissue microarrays of pancreatic adenocarcinoma were purchased from Shanghai Outdo Biotech Co., Ltd (Shanghai, China). Among them, three tissue microarrays from 174 patients had recorded follow‐up information. The median duration of patient follow‐up was 10.5 months (range 1–87 months). The other tissue microarray contained two normal pancreata, four cases of chronic pancreatitis, 13 pairs of malignant tumors and their matched adjacent noncancerous tissues, four metastatic tumors of the liver and abdominal wall, and eight positive metastatic lymph nodes.

### Immunohistochemistry

The tissue microarrays were deparaffinized and rehydrated at room temperature, and they were then immersed in methanol containing 0.3% hydrogen peroxide for 10 min to block endogenous peroxidase. Heat‐induced antigen retrieval was performed in a water bath for 30 min in a pH 6.0 antigen retrieval solution. After washing, the sections were incubated overnight with anti‐GPC1 antibody (1:200, Cat No. GTX104557; GeneTex Inc., Irvine, CA) at 4°C. The staining was performed using the Prolink‐2 Plus HRP rabbit polymer detection kit (Golden Bridge International Inc., Bothell, WA) according to the manufacturer's instructions. The images were captured using Aperio ScanScope CS software (Vista, CA).

The results were evaluated separately by two independent pathologists. The GPC1 staining intensity and area were quantified as described previously 14. Briefly, the GPC1 staining area was scored as follows: (1) 0, <5% of the epithelial cells in the respective lesions; (2) 1, 5–25% of the epithelial cells; (3) 2, 26–50% of the epithelial cells; (4) 3, 51–75% of the epithelial cells; and (5) 4, ≥75% of the epithelial cells. The intensity was graded as follows: (1) 0, negative; (2) 1+, weak (yellow); (3) 2+, moderate (light brown); and (4) 3+, strong (dark brown). A final score between 0 and 12 was achieved by multiplication of the extent of positivity and intensity. A staining index was used in which ≤1 was considered negative, 2–3 was weak, 4–7 was moderate, and ≥8 was considered strong expression.

### Statistical analysis

The Mann–Whitney *U* test or Kruskal–Wallis test was used to compare the read per million (RPM) values between two or multiple groups. In addition, the RPM value correlation matrix of six members from the glypican family was calculated by Spearman's rank correlation and visualized using the ggplot2 package in R (https://www.r-project.org/). The chi‐square test was used to compare qualitative data. The Kaplan–Meier method was used to determine the relationship between the levels of GPC1 and patient survival, and log‐rank analysis was performed to compare survival curves. Univariate and multivariate analyses were performed using the Cox regression model. *P* < 0.05 was considered significant. All analyses were performed using SPSS, version 19.0 (IBM software Inc., Chicago, IL).

## Results

### As the predominantly expressed glypican in pancreatic cancer, *GPC1* mRNA is associated with worse tumor biological characteristics

**Figure 1:**
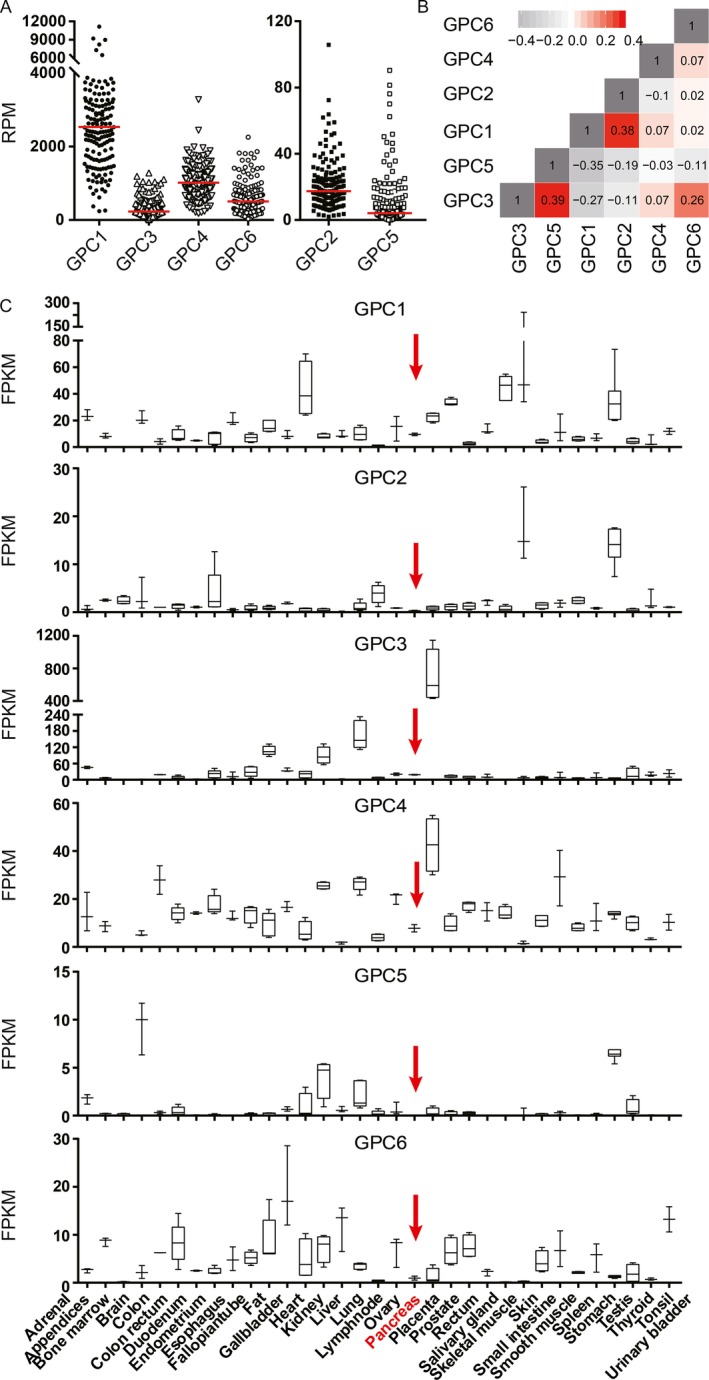
Gene expression of glypican family members in various tissues. (A) *GPC1* is the most strongly expressed glypicans in human PDAC tissues (*n* = 178) according to the TCGA RNA sequencing data. (B) The correlation matrix plot of six glypicans in PDAC tissues. Spearman's rank correlation coefficients were calculated and are shown in each square. (C) The tissue‐specific expression of glypican family members according to the normalized RNA‐sequencing data downloaded from the Human Protein Atlas portal (www.proteinatlas.org). The normal pancreas tissues are highlighted by red arrows.

**Figure 2:**
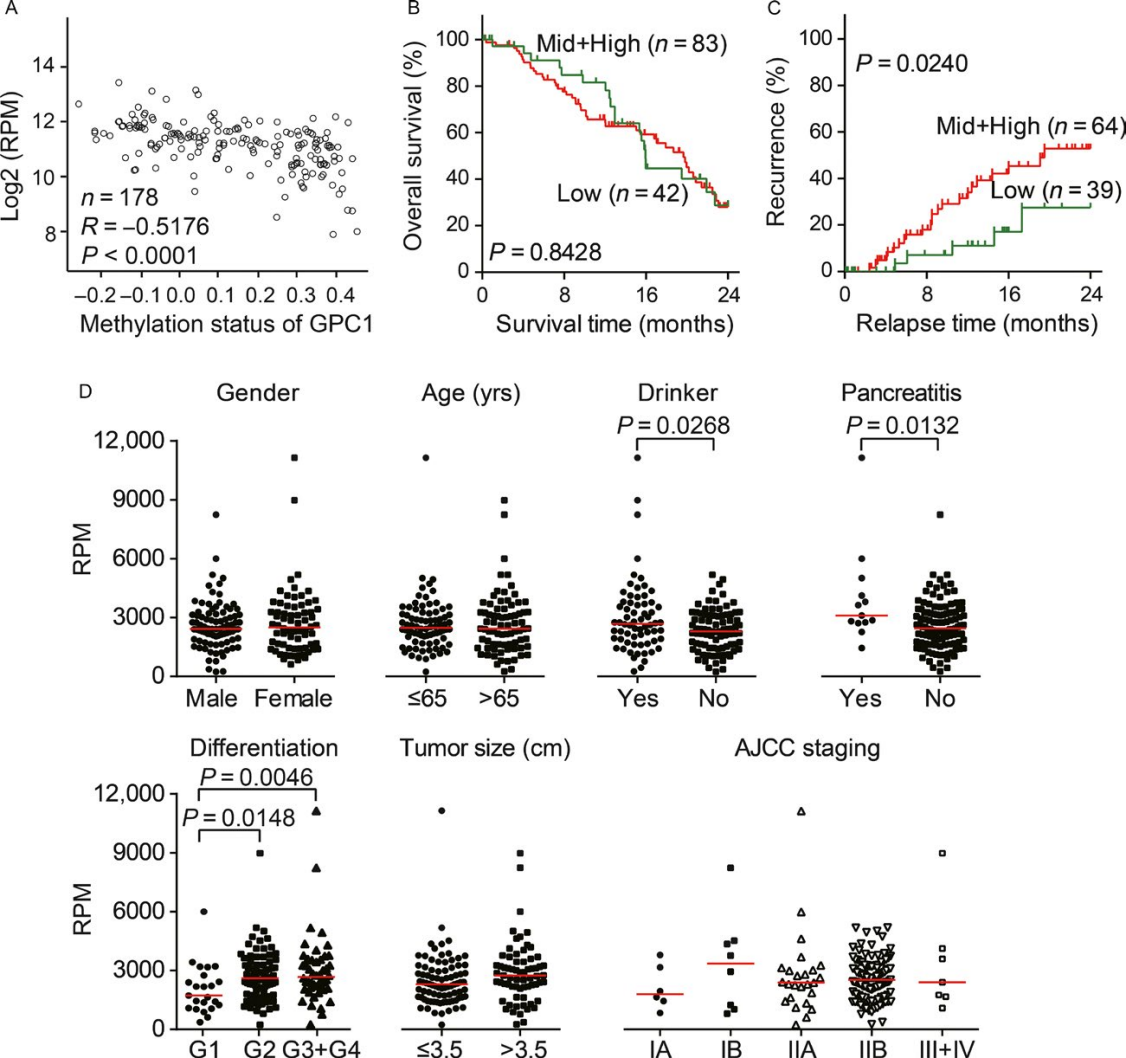
Clinicopathological characteristics of *GPC1* mRNA presented in the TCGA RNA sequencing data from pancreatic adenocarcinoma. (A) The expression of GPC1 mRNA was inversely correlated with DNA methylation levels in PDAC. Based on the TCGA RNA‐sequencing and DNA methylation 27k bead array datasets, 178 pancreatic cancer cases had both sets of data available. Pearson correlation coefficients were calculated between the log2 transformed read per million values and methylation status of GPC1. (B–C) Kaplan–Meier curve of overall survival (B) and recurrence‐free survival (C) according to the *GPC1* mRNA levels in PDAC tumor tissues. The cases were divided into two groups: a middle and high mRNA level group (upper 67th percentile) and a low mRNA level group (lower 33rd percentile). The log‐rank test was performed. (D) Clinicopathological characteristic analysis of *GPC1* mRNA expression in patients with pancreatic adenocarcinoma. The short red line represents the median value in each group.

According to the TCGA RNA sequencing data from pancreatic adenocarcinoma patients (*n* = 178), we observed that *GPC1* is the major expressed form in pancreatic adenocarcinoma among the six glypican family members in the human genome (GPC1‐6; Fig. 1A). Meanwhile, *GPC1* levels were negatively correlated with those of *GPC3* and *GPC5* and, positively correlated with *GPC2* (Spearman's rank correlation; Fig. 1B). Notably, the expression of glypicans is relatively tissue/cell‐specific based on the genome‐wide annotation of the tissue specificity of the human transcriptome (Fig. 1C), while normal pancreata barely express glypicans 15. Accordingly, *GPC1* was re‐expressed in pancreatic carcinogenesis. Promoter hypomethylation may be substantially responsible for the re‐expression of the *GPC1* gene because the methylation status of *GPC1* was inversely moderately correlated with its mRNA levels in pancreatic tumor tissues (Pearson correlation coefficient = −0.5176; *P *< 0.0001; Fig. 2A).

We divided the patients with PDAC into three groups, with high, moderate and low GPC1 expression levels, using two tertiles of RPM values of GPC1 in tumor tissues. Kaplan–Meier survival analysis with a log‐rank test showed that there was no correlation between *GPC1* mRNA levels and overall survival (Fig. 2B). However, moderate and high expression levels of GPC1 were significantly associated with a shorter relapse time (*P* = 0.0240; Fig. 2C).

The correlations between the clinicopathological characteristics of PDAC patients and *GPC1* mRNA expression in their tumors were also compared (Fig. 2D). The patients with alcoholic drinking habits, chronic pancreatitis, and poor pathological differentiation tended to express high levels of GPC1 (Mann–Whitney *U* tests, all of *P *< 0.05). Thus, it seems that GPC1 was ectopically expressed during tumorigenesis, and increased expression of GPC1 mRNA was associated with PDAC recurrence.

### GPC1 protein is absent in the exocrine portion of normal pancreata but is significantly overexpressed in PDAC

**Figure 3:**
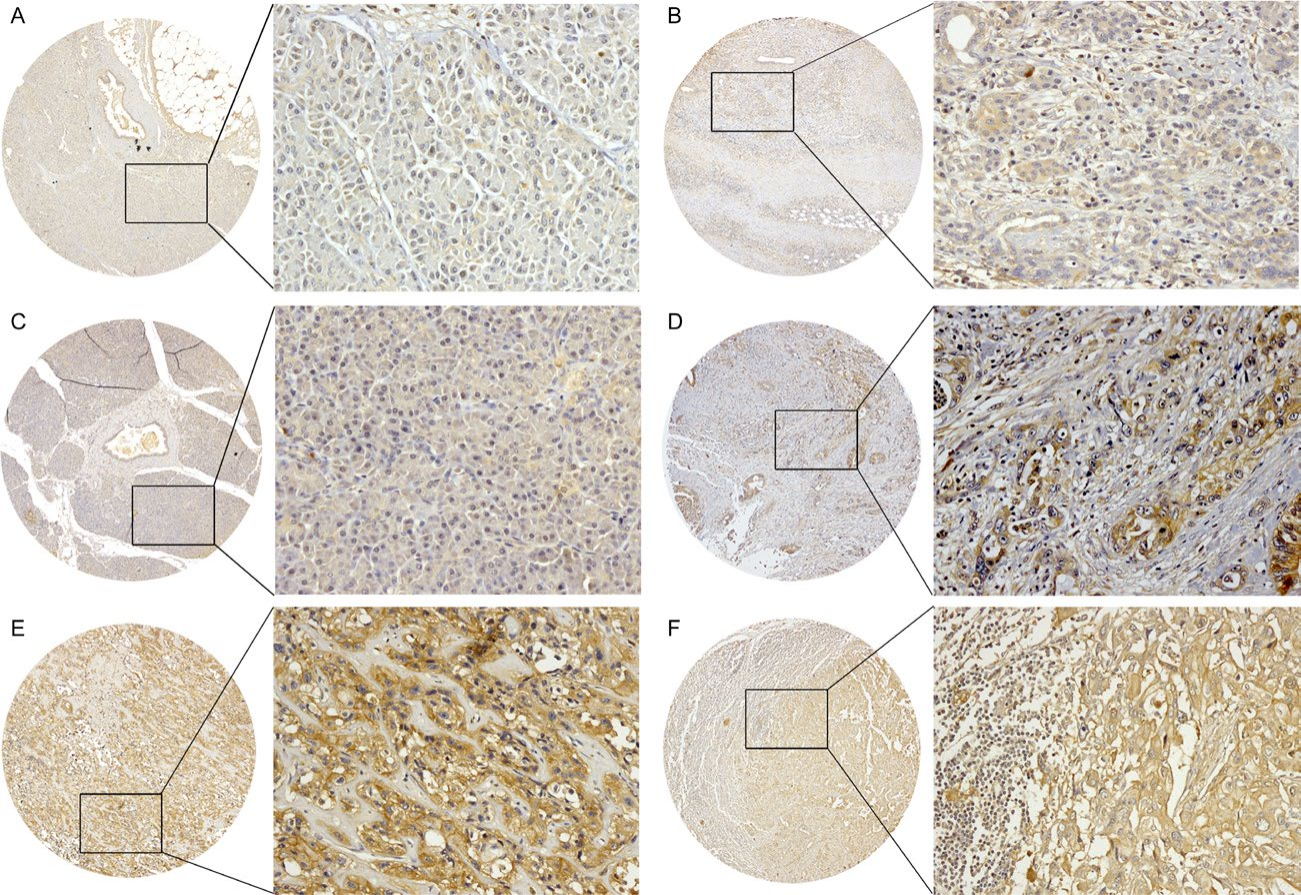
Representative immunohistochemistry images of GPC1 expression in normal pancreata (A), chronic pancreatitis (B), adjacent nontumor tissues (C), PDAC tumors (D), metastatic tumors in the liver (E), and metastatic lymph nodes (F). Original magnification: left panel, ×40; right panel, ×200.

**Figure 4:**
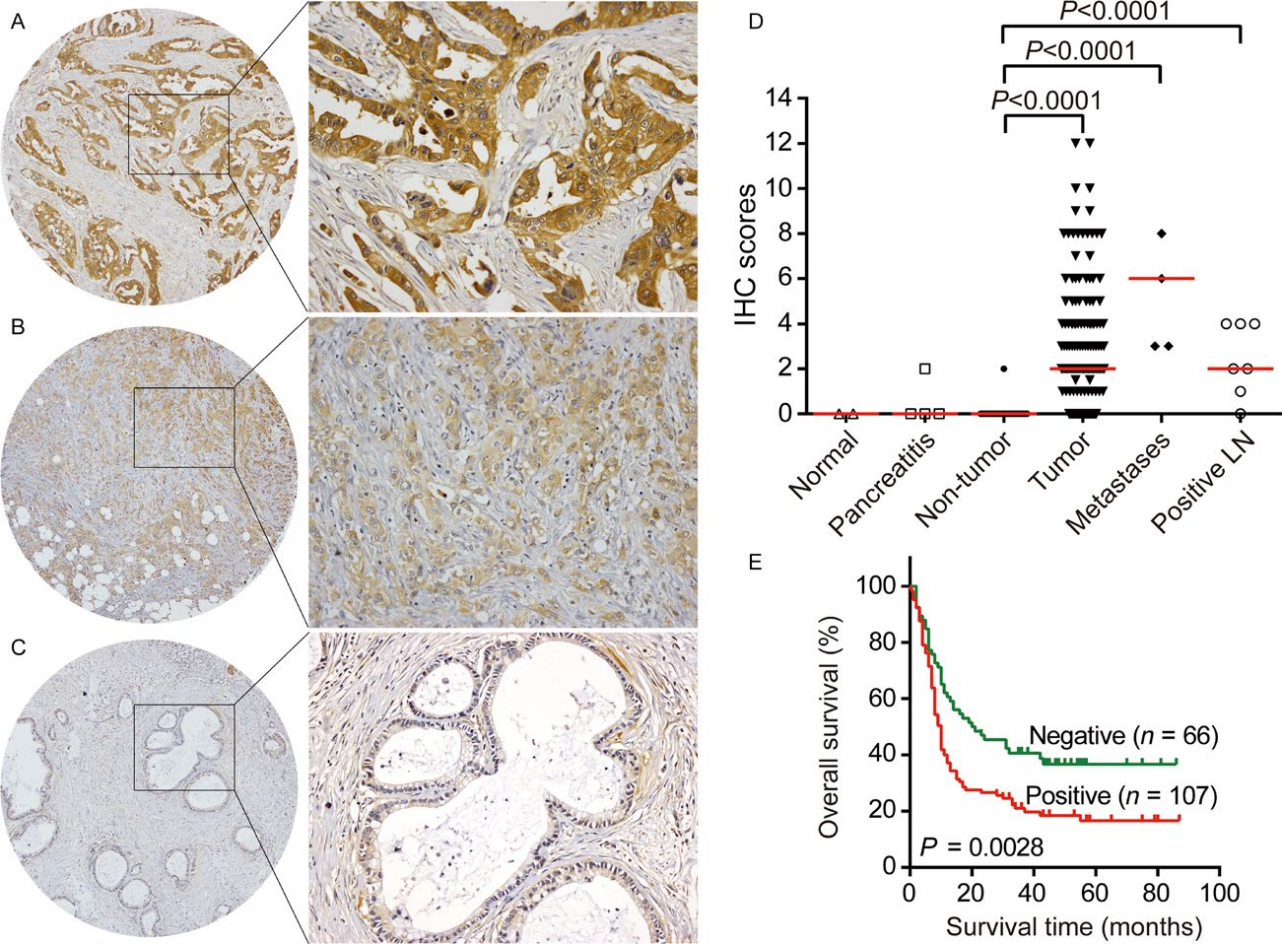
GPC1 protein expression was associated with poor prognosis in PDAC. Immunohistochemical staining shows strong (A), weak (B), and negative immunoreactivity to GPC1 in PDAC tumor tissues. Original magnification: left panel, ×40; right panel, ×200. (C) Distribution of GPC1 protein levels determined by immunohistochemistry in the normal pancreata (*n* = 2), chronic pancreatitis (*n* = 4), nontumor pancreatic tissues (*n* = 169), PDAC (*n* = 186), metastases (*n* = 4), and metastatic lymph nodes (*n* = 7). The short red line represents the median value in each group. (D) Kaplan–Meier curve of PDAC patients with negative and positive GPC1 expression. The log‐rank test was performed.

To confirm the aberrant expression of GPC1 at the transcriptional level, an immunohistochemistry assay was performed in normal pancreata (*n* = 2), chronic pancreatitis (*n* = 4), nontumor pancreata tissues (*n* = 169), PDAC (*n* = 186), metastases (*n* = 4) and metastatic lymph nodes (*n* = 7). GPC1 was undetectable in the normal pancreata (Fig. 3A). Most of the chronic pancreatitis cases still lacked GPC1 expression (Fig. 3B). In the adjacent noncancerous tissues, GPC1 staining remained barely detected (Fig. 3C), and only one specimen showed weak expression of GPC1 (0.6%, 1/169). Positive cytoplasmic and membrane immunostaining for GPC1 was observed in 59.7% (111/186) of the PDAC tumors (Table S1), 100% (4/4) of the liver or abdominal wall metastases, and 85.7% (6/7) of the metastatic lymph nodes (Fig. 3D–F). In the positively stained PDAC tissues, 57 (51.4%) had weak, 39 (35.1%) had moderate, and 15 (13.5%) had strong staining of GPC1 (Fig. 4A–C). Importantly, 35 of 55 cases (63.6%) with early‐stage PDAC (stage 1) showed positive staining of GPC1. In these 35 subjects, 57.1%, 31.4% and 11.4% showed weak, moderate and strong expression, respectively. Therefore, GPC1 is dramatically overexpressed in the PDAC and metastatic tissues compared with normal and benign pancreatic tissues, even in early‐stage patients (all *P *< 0.0001; Fig. 4D).

The correlations between the clinicopathological characteristics and GPC1 expression were subsequently analyzed (Table 1). Higher GPC1 levels were associated with being male (*P* = 0.0186) and with worse tumor biological features, including poor pathological differentiation (*P* = 0.0005) and larger tumor sizes (*P* = 0.0331). However, there was no correlation between GPC1 protein and tumor invasion depth, lymph node metastasis, and American Joint Committee on Cancer (AJCC) staging.

**Table 1:** The expression of GPC1 protein and its clinical significance in 186 PDAC specimens

	Total number of patients	Glypican‐1 staining		
	*n* = 186	Negative	Positive	*P* value^a^
Gender				0.0186
Male	118	40 (33.9)	78 (66.1)	
Female	68	35 (51.5)	33 (48.5)	
Age (year)				0.6274
≤60	79	30 (38.0)	49 (62.0)	
>60	106	44 (41.5)	62 (58.5)	
Differentiation				0.0005
I	17	14 (82.4)	3 (17.6)	
II	137	54 (39.4)	83 (60.6)	
III	22	5 (22.7)	17 (77.3)	
Tumor size (cm)				0.0331
≤6	160	70 (43.8)	90 (56.3)	
>6	24	5 (20.8)	19 (79.2)	
Nerve invasion				0.5749
No	107	45 (42.1)	62 (57.9)	
Yes	79	30 (38.0)	49 (62.0)	
Tumor depth				0.1705
T1, T2	107	41 (38.3)	66 (61.7)	
T3, T4	61	30 (49.2)	31 (50.8)	
Lymph node metastasis				0.5595
N0	102	41 (40.2)	61 (59.8)	
N1	74	33 (44.6)	41 (55.4)	
Distant organs metastasis				0.5684
M0	178	71 (39.9)	107 (60.1)	
M1	8	4 (50.0)	4 (50.0)	
AJCC staging				0.3032
Stage 1	55	20 (36.4)	35 (63.6)	
Stage 2, 3, 4	121	54 (44.6)	67 (55.4)	

a Chi‐square test was used.

### Prognostic relevance of GPC1 protein

Kaplan–Meier survival analysis indicated a significant correlation between positive GPC1 staining and a shorter overall survival time in the PDAC patients (*P* = 0.0028; Fig. 4E). The median survival time of the positive and negative expression groups was 10 and 20.5 months, respectively.

These findings were confirmed by both univariate and multivariate Cox regression analyses (Table 2). In the univariate analysis, the GPC1‐positive patients exhibited a 1.77‐fold increase in the hazard ratio (HR) for overall survival compared with the GPC1 negative group (*P* = 0.0028). The other significant risk factors included lymph node metastasis (*P* = 0.0019), distant metastasis (*P* = 0.0200) and AJCC staging (*P* = 0.0004). In the multivariate analysis, GPC1 expression (HR = 1.82, *P* = 0.0022) and AJCC staging (HR = 2.24, *P* = 0.0002) were independent prognostic factors for mortality.

**Table 2:** Univariate and multivariate survival analysis of GPC1 expression for overall survival in 186 patients with PDAC

	Hazard ratio (95% CI)	*P* value
Univariate		
Gender (female vs. male)	0.81 (0.57–1.17)	0.2639
Age (>60 vs. ≤60 years old)	1.23 (0.87–1.74)	0.2468
Differentiation (Poorly vs. moderately vs. well)	1.41 (0.94–2.13)	0.0987
Tumor size (>6 cm vs. ≤ 6 cm)	1.16 (0.70–1.94)	0.5624
Nerve invasion (yes vs. no)	1.08(0.76–1.53)	0.6706
Tumor invasive depth (T4 + T3 vs. T1 + T2)	1.25 (0.85–1.83)	0.2584
Lymph node metastasis (N1b, c+N2 vs. N0 + N1a)	1.78 (1.24–2.56)	0.0019
Distant organs metastasis (M1 vs. M0)	2.70 (1.17–6.10)	0.0200
AJCC staging (IV + III + II vs. I)	2.15 (1.40–3.29)	0.0004
Glypican‐1 expression (positive vs. negative)	1.77 (1.22–2.57)	0.0028
Multivariate		
AJCC staging (IV + III + II vs. I)	2.24 (1.47–3.43)	0.0002
Glypican‐1 expression (positive vs. negative)	1.82 (1.24–2.67)	0.0022

## Discussion

In general, the expression of glypicans is regulated in a developmental stage‐ and tissue‐specific manner. However, in this study, we observed abnormal expression of GPC1 mRNA and protein in pancreatic cancers, in agreement with previous reports 12, 13. One of the most important findings in this study is the observation that promoter hypomethylation is responsible for the re‐expression of the *GPC1* gene to a great extent. Based on the TCGA samples (*n* = 178), hypomethylation could moderately explain its overexpression, with an R‐squared value of 0.2679. In addition, gene amplification may also result in its aberrant expression. Although the amplification event was observed in only one case among the TCGA samples (1/185), the *GPC1* gene amplification rate reached 11% (12/109) in another whole‐exome sequencing sample set with PDAC 16. This result suggests that promoter hypomethylation and gene amplification of *GPC1* are frequent in PDAC.

In addition, we found that GPC1 expression was associated with worse biological behaviors of PDAC, such as poorer differentiation and larger tumor diameters, indicating that aberrantly expressed GPC1 may play important roles in tumorigenesis. Previous studies had shown that GPC1 strengthened the mitogenic responses of PDAC cells to fibroblast growth factor 2 (FGF2), heparin binding EGF‐like growth factor (HB‐EGF), and hepatocyte growth factor (HGF) as a growth factor coreceptor 12, 17, 18. It also enhanced the deleterious actions of TGF‐*β* in pancreatic cancer cells 19, 20. Accordingly, GPC1 is crucial for efficient growth, metastasis, and angiogenesis in cancer cells and genetic mouse models of PDAC 21, 22. Therefore, it is understandable that GPC1 is an independent unfavorable prognostic factor for PDAC. Furthermore, at the mRNA level, we found that GPC1 was higher in patients with a chronic pancreatitis background and drinking habits, indicating that inflammation‐related risk factors may facilitate the expression of GPC1 in PDAC. We suspect that the clinical significance of GPC1 is not entirely consistent at the mRNA and protein levels due to the different specimens used and the differential abundance between mRNA and protein. In addition, PDAC is one of the most stroma‐rich cancers 23. The heterogeneous stroma is composed of various cellular and extracellular components, including mesenchymal cells, extracellular matrix (ECM), and soluble proteins. Therefore, a correlation analysis of GPC1 mRNA and protein levels needs to be performed with the same batch of microdissected tissue specimens to exclude the influence of ECM and stromal components.

During early neurogenesis, GPC1 determines brain size and the differentiation of neural stem cells by regulating FGF and canonical Wnt signaling 24, 25, 26. In commissural neurons, GPC1 is required as a coreceptor for the Sonic Hedgehog (Shh)‐dependent induction of its own receptor to mediate commissural axon guidance 27. GPC1 could also act as a negative regulator of Shh signaling in biliary development, and downregulation of GPC1 led to developmental biliary defects and biliary atresia 28. In bone, GPC1 regulates bone morphogenetic protein 2 (BMP2)‐mediated osteogenesis 29. In endothelial cells, GPC1 induces metaphase arrest and centrosome overproduction, and it mediates flow‐induced NO synthase (eNOS) activation to protect endothelial function 30, 31, 32. Because of the essential roles of GPC1 in the pathways of heparin binding growth factors and the TGF‐*β* families, Wnt and Shh, it appears that GPC1 participates in the carcinogenesis of several tumors. Except for PDAC, the overexpression of GPC1 has been observed in the tumor tissues of gliomas, ameloblastomas, prostate cancer and esophageal squamous cell carcinoma (ESCC) 7, 33, 34, 35, 36, 37. However, contradictory results were observed in colorectal cancer and breast cancer 38, 39, 40, 41. In ESCC, high levels of GPC1 were also significantly associated with chemoresistance to cisplatin 33.

Importantly, GPC1 was regarded as a more promising biomarker for PDAC. In tissues, our study and previous studies showed that GPC1 was dramatically overexpressed in malignant cells 12, 13. In serum, circulating GPC1 showed a similar diagnostic performance to CA19‐9; however, the sensitivity and specificity of GPC1^+^ crExos were greater than those of soluble GPC1. Notably, GPC1^+^ crExos was a near perfect classifier in distinguishing patients with PDAC from healthy donors and patients with benign pancreatic disease, even for early‐stage PDAC 7. Moreover, compared with levels in breast cancer patients, GPC1^+^ crExos was higher in PDAC patients. Notably, for either mRNA or protein, our results revealed that the increase in GPC1 in malignant cells was independent of AJCC stage, suggesting that it is an excellent candidate for early‐stage PDAC. It seems that GPC1‐based measurement will aid in early diagnosis and provide more opportunities for curative surgical therapy for PDAC patients. Therefore, our results further support the notion that GPC1 is an attractive diagnostic and prognostic biomarker for detecting early stages of pancreatic cancer.

In summary, we investigated the mRNA and protein expression features of GPC1 in large‐scale clinical samples of PDAC. The results demonstrated that the *GPC1* gene was re‐expressed in PDAC mainly due to promoter hypomethylation, even for early‐stage PDAC. High levels of GPC1 were associated with poorer pathological differentiation and worse biological behaviors. GPC1 could serve as an independent unfavorable prognostic factor in PDAC. Further studies in a larger cohort will be required for unequivocal validation of its clinical application, especially for very early stages of the disease. However, given the oncogenic roles of GPC1, GPC1 is indeed a powerful diagnostic and prognostic marker as well as a promising therapeutic target for PDAC.

## Conflict of Interest

The authors have no conflicts of interest to declare.

## Supporting information


**Table S1.** The immunohistochemical staining results of 186 PDAC specimens.
