# Supplementary material for: Elevated glypican‐1 expression is associated with an unfavorable prognosis in pancreatic ductal adenocarcinoma
Source: Cancer Med. 2017 Apr 24;6(6):1181–91. doi: 10.1002/cam4.1064 (PMC5463070; doi:10.1002/cam4.1064)
Supplement: Supplementary file 1 — Table S1. The immunohistochemical staining results of 186 PDAC specimens. [file CAM4-6-1181-s001.doc]

**Supporting information**

**Elevated glypican-1 expression is associated with an unfavorable prognosis in pancreatic ductal adenocarcinoma**

Haizhen Lu^1^, Fangfei Niu^2^, Fang Liu^2^, Jiajia Gao^2^, Yulin Sun^2^, Xiaohang Zhao^2^

^1^Department of Pathology, National Cancer Center/Cancer Hospital, Chinese Academy of Medical Science & Peking Union Medical College, Beijing 100021, China

^2^State Key Laboratory of Molecular Oncology, National Cancer Center/Cancer Hospital, Chinese Academy of Medical Sciences & Peking Union Medical College, Beijing 100021, China

**Supplementary Table S1.** The immunohistochemical staining results of 186 PDAC specimens

|  | **Intensity scores** | **Staining area scores** | **Intensity × area scores** | **Staining index** |
| --- | --- | --- | --- | --- |
| Sample 1 | 2 | 2 | 4 | Moderate |
| Sample 2 | 1 | 1 | 1 | Negative |
| Sample 3 | 2 | 1 | 2 | Weak |
| Sample 4 | 2 | 2 | 4 | Moderate |
| Sample 5 | 0 | 0 | 0 | Negative |
| Sample 6 | 2 | 3 | 6 | Strong |
| Sample 7 | 2 | 1 | 2 | Weak |
| Sample 8 | 2 | 2 | 4 | Moderate |
| Sample 9 | 0 | 0 | 0 | Negative |
| Sample 10 | 0 | 0 | 0 | Negative |
| Sample 11 | 0 | 0 | 0 | Negative |
| Sample 12 | 0 | 0 | 0 | Negative |
| Sample 13 | 3 | 1 | 3 | Weak |
| Sample 14 | 3 | 3 | 9 | Strong |
| Sample 15 | 3 | 1 | 3 | Weak |
| Sample 16 | 2 | 2 | 4 | Moderate |
| Sample 17 | 2 | 2 | 4 | Moderate |
| Sample 18 | 1 | 3 | 3 | Weak |
| Sample 19 | 1 | 4 | 4 | Moderate |
| Sample 20 | 2 | 2 | 4 | Moderate |
| Sample 21 | 0 | 0 | 0 | Negative |
| Sample 22 | 0 | 0 | 0 | Negative |
| Sample 23 | 2 | 2 | 4 | Moderate |
| Sample 24 | 2 | 2 | 4 | Moderate |
| Sample 25 | 0 | 0 | 0 | Negative |
| Sample 26 | 0 | 0 | 0 | Negative |
| Sample 27 | 2 | 1 | 2 | Weak |
| Sample 28 | 1 | 2 | 2 | Weak |
| Sample 29 | 2 | 2 | 4 | Moderate |
| Sample 30 | 3 | 1 | 3 | Weak |
| Sample 31 | 2 | 2 | 4 | Moderate |
| Sample 32 | 2 | 3 | 6 | Moderate |
| Sample 33 | 1 | 3 | 3 | Weak |
| Sample 34 | 0 | 0 | 0 | Negative |
| Sample 35 | 0 | 0 | 0 | Negative |
| Sample 36 | 1 | 3 | 3 | Weak |
| Sample 37 | 1 | 1 | 1 | Negative |
| Sample 38 | 1 | 1 | 1 | Negative |
| Sample 39 | 1 | 2 | 2 | Weak |
| Sample 40 | 0 | 0 | 0 | Negative |
| Sample 41 | 0 | 0 | 0 | Negative |
| Sample 42 | 1 | 2 | 2 | Weak |
| Sample 43 | 1 | 2 | 2 | Weak |
| Sample 44 | 2 | 2 | 4 | Moderate |
| Sample 45 | 0 | 0 | 0 | Negative |
| Sample 46 | 0 | 0 | 0 | Negative |
| Sample 47 | 2 | 2 | 4 | Moderate |
| Sample 48 | 2 | 2 | 4 | Moderate |
| Sample 49 | 0 | 0 | 0 | Negative |
| Sample 50 | 3 | 3 | 9 | Moderate |
| Sample 51 | 0 | 0 | 0 | Negative |
| Sample 52 | 2 | 2 | 4 | Moderate |
| Sample 53 | 0 | 0 | 0 | Negative |
| Sample 54 | 0 | 0 | 0 | Negative |
| Sample 55 | 1 | 3 | 3 | Weak |
| Sample 56 | 0 | 0 | 0 | Negative |
| Sample 57 | 2 | 2 | 4 | Weak |
| Sample 58 | 1 | 1 | 1 | Negative |
| Sample 59 | 0 | 0 | 0 | Negative |
| Sample 60 | 2 | 2 | 4 | Moderate |
| Sample 61 | 0 | 0 | 0 | Negative |
| Sample 62 | 2 | 2 | 4 | Weak |
| Sample 63 | 0 | 0 | 0 | Negative |
| Sample 64 | 2 | 2 | 4 | Moderate |
| Sample 65 | 3 | 2 | 6 | Moderate |
| Sample 66 | 2 | 3 | 6 | Moderate |
| Sample 67 | 0 | 0 | 0 | Negative |
| Sample 68 | 0 | 0 | 0 | Negative |
| Sample 69 | 1 | 3 | 3 | Weak |
| Sample 70 | 1 | 2 | 2 | Weak |
| Sample 71 | 1 | 2 | 2 | Weak |
| Sample 72 | 0 | 0 | 0 | Negative |
| Sample 73 | 3 | 3 | 9 | Strong |
| Sample 74 | 2 | 3 | 6 | Moderate |
| Sample 75 | 2 | 2 | 4 | Weak |
| Sample 76 | 1 | 1 | 1 | Negative |
| Sample 77 | 3 | 3 | 9 | Strong |
| Sample 78 | 1 | 1 | 1 | Negative |
| Sample 79 | 1 | 2 | 2 | Weak |
| Sample 80 | 0 | 0 | 0 | Negative |
| Sample 81 | 2 | 3 | 6 | Moderate |
| Sample 82 | 1 | 1 | 1 | Negative |
| Sample 83 | 0 | 0 | 0 | Negative |
| Sample 84 | 0 | 0 | 0 | Negative |
| Sample 85 | 3 | 3 | 9 | Strong |
| Sample 86 | 2 | 4 | 8 | Strong |
| Sample 87 | 2 | 1 | 2 | Weak |
| Sample 88 | 0 | 0 | 0 | Negative |
| Sample 89 | 1 | 3 | 3 | Weak |
| Sample 90 | 2 | 1 | 2 | Weak |
| Sample 91 | 0 | 0 | 0 | Negative |
| Sample 92 | 2 | 2 | 4 | Moderate |
| Sample 93 | 2 | 2 | 4 | Moderate |
| Sample 94 | 2 | 2 | 4 | Moderate |
| Sample 95 | 0 | 0 | 0 | Negative |
| Sample 96 | 0 | 0 | 0 | Negative |
| Sample 97 | 0 | 0 | 0 | Negative |
| Sample 98 | 0 | 0 | 0 | Negative |
| Sample 99 | 3 | 4 | 12 | Strong |
| Sample 100 | 1 | 4 | 4 | Moderate |
| Sample 101 | 1 | 3 | 3 | Weak |
| Sample 102 | 1 | 2 | 2 | Weak |
| Sample 103 | 3 | 4 | 12 | Strong |
| Sample 104 | 1 | 2 | 2 | Weak |
| Sample 105 | 0 | 0 | 0 | Negative |
| Sample 106 | 0 | 0 | 0 | Negative |
| Sample 107 | 0 | 0 | 0 | Negative |
| Sample 108 | 1 | 2 | 2 | Weak |
| Sample 109 | 2 | 3 | 6 | Moderate |
| Sample 110 | 0 | 0 | 0 | Negative |
| Sample 111 | 1 | 1 | 1 | Negative |
| Sample 112 | 1 | 2 | 2 | Weak |
| Sample 113 | 0 | 0 | 0 | Negative |
| Sample 114 | 1 | 4 | 4 | Moderate |
| Sample 115 | 1 | 1 | 1 | Negative |
| Sample 116 | 1 | 2 | 2 | Weak |
| Sample 117 | 1 | 2 | 2 | Weak |
| Sample 118 | 0 | 0 | 0 | Negative |
| Sample 119 | 2 | 1 | 2 | Weak |
| Sample 120 | 2 | 2 | 4 | Moderate |
| Sample 121 | 0 | 0 | 0 | Negative |
| Sample 122 | 0 | 0 | 0 | Negative |
| Sample 123 | 1 | 3 | 3 | Weak |
| Sample 124 | 0 | 0 | 0 | Negative |
| Sample 125 | 0 | 0 | 0 | Negative |
| Sample 126 | 1 | 2 | 2 | Weak |
| Sample 127 | 2 | 4 | 8 | Strong |
| Sample 128 | 2 | 4 | 8 | Strong |
| Sample 129 | 1 | 2 | 2 | Weak |
| Sample 130 | 0 | 0 | 0 | Negative |
| Sample 131 | 2 | 1 | 2 | Weak |
| Sample 132 | 2 | 3 | 6 | Moderate |
| Sample 133 | 1 | 4 | 4 | Moderate |
| Sample 134 | 1 | 2 | 2 | Weak |
| Sample 135 | 0 | 0 | 0 | Negative |
| Sample 136 | 0 | 0 | 0 | Negative |
| Sample 137 | 2 | 3 | 6 | Moderate |
| Sample 138 | 2 | 4 | 8 | Strong |
| Sample 139 | 0 | 0 | 0 | Negative |
| Sample 140 | 3 | 1 | 3 | Weak |
| Sample 141 | 0 | 0 | 0 | Negative |
| Sample 142 | 1 | 4 | 4 | Moderate |
| Sample 143 | 1 | 1 | 1 | Negative |
| Sample 144 | 1 | 2 | 2 | Weak |
| Sample 145 | 1 | 3 | 3 | Weak |
| Sample 146 | 3 | 4 | 12 | Strong |
| Sample 147 | 2 | 4 | 8 | Strong |
| Sample 148 | 1 | 2 | 2 | Weak |
| Sample 149 | 1 | 3 | 3 | Weak |
| Sample 150 | 1 | 1 | 1 | Negative |
| Sample 151 | 1 | 3 | 3 | Weak |
| Sample 152 | 1 | 2 | 2 | Weak |
| Sample 153 | 2 | 1 | 2 | Weak |
| Sample 154 | 3 | 2 | 6 | Moderate |
| Sample 155 | 1 | 2 | 2 | Weak |
| Sample 156 | 0 | 0 | 0 | Negative |
| Sample 157 | 1 | 1 | 1 | Negative |
| Sample 158 | 1 | 2 | 2 | Weak |
| Sample 159 | 1 | 2 | 2 | Weak |
| Sample 160 | 2 | 3 | 6 | Moderate |
| Sample 161 | 0 | 0 | 0 | Negative |
| Sample 162 | 2 | 3 | 6 | Moderate |
| Sample 163 | 1 | 2 | 2 | Weak |
| Sample 164 | 2 | 1 | 2 | Weak |
| Sample 165 | 2 | 1 | 2 | Weak |
| Sample 166 | 0 | 0 | 0 | Negative |
| Sample 167 | 2 | 1 | 2 | Weak |
| Sample 168 | 1 | 2 | 2 | Weak |
| Sample 169 | 3 | 1 | 3 | Weak |
| Sample 170 | 1 | 2 | 2 | Weak |
| Sample 171 | 2 | 3 | 6 | Moderate |
| Sample 172 | 1 | 1 | 1 | Negative |
| Sample 173 | 2 | 4 | 8 | Strong |
| Sample 174 | 1 | 2 | 2 | Weak |
| Sample 175 | 2 | 4 | 8 | Strong |
| Sample 176 | 1 | 1 | 1 | Negative |
| Sample 177 | 1 | 1 | 1 | Negative |
| Sample 178 | 0 | 0 | 0 | Negative |
| Sample 179 | 1 | 2 | 2 | Weak |
| Sample 180 | 2 | 3 | 6 | Moderate |
| Sample 181 | 0 | 0 | 0 | Negative |
| Sample 182 | 0 | 0 | 0 | Negative |
| Sample 183 | 0 | 0 | 0 | Negative |
| Sample 184 | 0 | 0 | 0 | Negative |
| Sample 185 | 0 | 0 | 0 | Negative |
| Sample 186 | 0 | 0 | 0 | Negative |
